# 2322 Modulation of autophagy in intestinal health and inflammation

**DOI:** 10.1017/cts.2018.94

**Published:** 2018-11-21

**Authors:** Eliseo Castillo

**Affiliations:** Clinical and Translational Science Center, University of New Mexico

## Abstract

OBJECTIVES/SPECIFIC AIMS: Modulation of autophagy has the potential to treat inflammatory bowel disease (IBD). IBD is characterized by dysregulated inflammatory pathways and a defective intestinal epithelial barrier. We sought to better understand how autophagy can be utilized to regulate both inflammation and the intestinal barrier. METHODS/STUDY POPULATION: We examined mice with an autophagy defect in only macrophages in an animal model of IBD. To understand the phenotype, we utilized macrophages to investigate the mechanism behind autophagy and proinflammatory cytokine secretion. In addition, we analyzed the development of colonoids in a co-culture system with macrophages with or without a functional autophagy pathway. Lastly, pharmacological modulation of autophagy to control inflammation was assessed. RESULTS/ANTICIPATED RESULTS: Mice with autophagy-deficient macrophages were highly susceptible to intestinal barrier disruption. Susceptibility was due to enhanced proinflammatory cytokine secretion and intestinal permeability. Furthermore, proinflammatory macrophages (due to an autophagy defect) co-cultured with colonoids, significantly decreased the number of mucus producing goblet cells. Finally, pharmacologically modulation of autophagy reduced the secretion of proinflammatory cytokine by macrophages and reduced intestinal permeability. DISCUSSION/SIGNIFICANCE OF IMPACT: Our results strongly suggest autophagy modulation can dampen inflammation and enhance the intestinal epithelial barrier.

